# Age-Related Differences in Naming Latency Variability

**DOI:** 10.5334/joc.520

**Published:** 2026-09-21

**Authors:** Sabia Costantini, Pamela Fuhrmeister, Audrey Bürki, Leonie F. Lampe

**Affiliations:** 1 Cognitive Science, University of Potsdam, Germany; 2 Linguistic Research Infrastructure, University of Zürich, Switzerland

**Keywords:** Variability, Naming Latencies, Aging

## Abstract

Variability in naming latencies is expected to increase with age on multiple levels, but few studies have examined whether this is indeed the case. In the present study, we tested whether performance variability increases with age by comparing young and older adults in the amount of naming latency variability between participants, across items, and within participants across trials and days. We additionally used estimates of performance variability for power analyses to determine how samples (i.e., participants/items) should be adjusted to achieve sufficient power in studies comparing latencies across age groups. We tested older adults (aged 65–75) on two occasions (7–16 days apart) and compared their naming latencies with those of young adults (aged 18–35) tested in another study using the same procedures. We replicated the age-related slowing in naming latencies reported by some authors. We found greater between-item variability in older participants, no evidence for or against an age-related increase in between-participant variability, and evidence for no increases in participant variability across trials with age. Moreover, variability across days was greater in the young than the older adult group. We discuss the relevance of these findings for designing future studies as well as for better understanding mixed findings in past studies.

## Introduction

Several cognitive abilities have been shown to decline with age. In the domain of language, one ability that has been argued to become less efficient is word retrieval, the process through which words stored in long-term memory are accessed for production. Studies show, for instance, that older adults report more tip-of-the-tongue episodes—temporary failures to retrieve known words (Burke, MacKay, Worthley, & Wade, 1991; Heine, Ober, & Shenaut, 1999)—and tend to retrieve fewer words than younger adults in word fluency tasks (Baciu et al., 2016; Brickman et al., 2005; Meinzer et al., 2009). Moreover, several studies have found that when asked to name pictures of common nouns, older adults tend to need more time to plan their response, as measured with the time interval between the onset of picture presentation and the initiation of the vocal response, or naming latency (Ferré, Jarret, Brambati, Bellec, & Joanette, 2020; Hoyau et al., 2017; Krethlow, Fargier, Atanasova, Ménétré, & Laganaro, 2024; Oosterhuis, Slade, Smith, May, & Nuttall, 2023; Tsang & Lee, 2003; Verhaegen & Poncelet, 2013; Wierenga et al., 2008). Studies have capitalized on the age difference in naming latencies to try better understand the cognitive mechanisms underlying changes in word retrieval with age (e.g., Taylor & Burke, 2002).

In the present study, we extend on those findings to examine changes in the variability of naming latencies with age. There are reasons to expect more variability in older adults’ naming performance compared to young adults. First, we can expect the age-related decline in word retrieval to differ across individuals, leading to considerable between-participant variability in older adults’ naming latencies. Studies have indeed reported a larger variability in naming latencies across older than younger speakers (e.g., Ferré et al., 2020; Oosterhuis et al, 2023; Tsang & Lee, 2003) but note that these differences were not tested statistically. Outside the area of language processing, numerous studies highlight major inter-individual differences in aging trajectories (e.g., Abrous et al., 2026), and there are no reasons to expect that word retrieval decline affects individuals in a more homogeneous way.

Second, older adults can be expected to show a larger between-item variability. Studies have indeed reported that older adults tend to show larger effects of item properties on naming latencies compared to young adults. This has been found for various item properties, such as name agreement (LaGrone & Spieler, 2006) and word frequency (Spieler & Balota, 2000).

Finally, naming latencies of older individuals can be expected to be more variable across trials and/or across days. Greater within-participant variability (from trial to trial or across days) is expected under the assumption that naming requires executive functions. This prediction is based on the findings that performance variability tends to increase with age in tasks that rely on executive functions (see West, Murphy, Armilio, Craik, & Stuss, 2002).

To summarize, the naming latencies of older adults can be expected to be more variable between participants, between items and within participants. Surprisingly, few studies have tested whether this is indeed the case. Information about variability is useful for several reasons. First, estimates of variability can be used to run power analyses to determine the number of participants and items needed to achieve sufficient power in studies comparing naming latencies across age groups. Second, information about variability can provide new insights into previous findings on naming performance in aging. The magnitude of the age-related difference in naming latencies varies considerably across investigations. For example, Tsang and Lee (2003) observed a difference of 1210 ms, while Krethlow et al. (2024) reported a much smaller effect size (i.e., 109 ms). Moreover, this difference is not always replicated (Baciu et al., 2016; Evrard, 2002; Hoyau et al., 2018; Valente & Laganaro, 2015). Finally, information on sources of variability can inform theories of aging in language production.

The aim of the present study is precisely to assess the extent of variability in picture naming latency in older individuals. To do so, we tested a group of older adults in a picture naming experiment at two different time points and compared their performance to that of a group of younger adults undergoing an identical procedure (Fuhrmeister, Elbuy, & Bürki, 2024). We structure our work in the following manner: First, we test whether we can replicate the age-related slowing reported by some authors and estimate the size of this effect. Second, we estimate and compare different types of variability across age groups, that is, between participants, across items, and within participants across trials and days. Finally, we ran power calculations to determine the number of participants and items needed to achieve sufficient power in naming studies with age group comparisons.

## Methods

### Participants

Eighty-two adults were tested through the online platform Prolific (www.prolific.com). Participants were aged between 65 and 75 years (mean = 69.15, SD = 2.92) and reported no language-related disorders, color-blindness, or a diagnosis of cognitive impairment. All participants spoke English as their first language and resided in the United Kingdom. Participants were excluded if their data had not been properly recorded (n = 12), if the quality of the vocal recordings was poor (n = 8), if they did not meet the study inclusion criteria (n = 2) or if they did not complete both sessions (n = 2). We additionally sought to exclude participants if their accuracy score on the naming task on both sessions was below 60%. However, all participants performed above this threshold. We finally excluded participants (n = 4) who scored two standard deviations above the group’s mean on the Cognitive Failure Questionnaire 2.0 (Goodhew & Edwards, 2024, see next section). We did not perform any descriptive or inferential statistics prior to removing these participants. The final dataset for older adults included 54 participants. Participants gave informed consent prior to the experiment and received monetary compensation for their participation (€11/h). This study was approved by the ethics board of the University of Potsdam.

Young adults’ data were taken from a previous study conducted in our lab (Fuhrmeister et al., 2024). This dataset included 50 young adults aged between 18 and 35 (mean = 27.8, SD = 4.59). Participants did not report any hearing or language disorders. As with the older adult group, all participants were native English speakers and resided in the United Kingdom. Participants gave informed consent prior to the experiment and received monetary compensation for their participation (€11/h). The study was approved by the ethics board of the University of Potsdam.

### Tasks and stimuli

#### Picture naming task

To ensure comparability between young and older adults’ performance, we used the same experimental material used in Fuhrmeister et al. (2024). The stimuli consisted of 310 colored pictures from the Multipic database (Duñabeitia et al., 2018) depicting common nouns (e.g., objects, animals, professions, body parts, food items). The stimuli were divided into two experimental lists, each consisting of 155 pictures. Lists were balanced on word frequency, name agreement, and age of acquisition, as these variables have been found to be robust predictors of naming latencies during naming tasks (Alario et al., 2004; Perret & Bonin, 2019). Lists can be found in Appendix A.

Fuhrmeister and colleagues balanced the lists in the following manner. The authors selected pictures with high name agreement from the Multipic database (maximum H-index of 0.52). They obtained values for name agreement, frequency, and age of acquisition for all pictures and z-scored them. These values were used to form a feature vector for each stimulus. The authors calculated the cosine similarity of the feature vectors for each unique pair of stimuli and sampled one million random sets of 155 item pairings. Subsequently, they calculated the mean cosine similarity of all the pairings in each set and selected the set of 155 pairings with the highest mean cosine similarity for the two lists, as these were the most similar in terms of lexical frequency, name agreement, and age of acquisition (cosine similarity = 0.24). Descriptive statistics on the variables of interest for each list can be found in Table 1. Reproducible code for list creation can be found at the OSF repository of Fuhrmeister et al. (2024): https://osf.io/jqmtv/.

**Table 1:** Mean and standard deviation (SD) of frequency (Zipf scale), age of acquisition (AoA) ratings, and name agreement (H-index) for the words/pictures in each list.


LIST	FREQUENCY MEAN	FREQUENCY SD	AOA MEAN	AOA SD	H-INDEX MEAN	H-INDEX SD

1	4.17	0.48	5.39	1.43	0.13	0.15

2	4.28	0.64	5.48	1.69	0.13	0.17


Participants saw one of the two lists of pictures at each session (5 pictures per list for training items; 150 pictures per list for test items). The order of list presentation was counterbalanced (i.e., half of the participants saw list 1 for session 1 and half saw list 1 for session 2). All participants saw the same 5 pictures in each list for training.

#### Cognitive Failure Questionnaire 2.0

We used the Cognitive Failure Questionnaire 2.0 (Goodhew & Edwards, 2024) as a screening tool to exclude participants whose data were above group performance regarding everyday cognitive failures. We used the Cognitive Failure Questionnaire 2.0 rather than the original version of the test (i.e., Cognitive Failure Questionnaire, Broadbent, Cooper, FitzGerald, & Parkes, 1982) as the updated questionnaire is shorter and has a higher contemporary relevance. The Cognitive Failure Questionnaire 2.0 consists of fifteen questions assessing how frequently respondents have experienced specific cognitive failures over the past six months, such as forgetting passwords (question 8) or calling people by the wrong name (question 10). Answers are given on a five-point Likert scale (0 = never; 4 = very often). High scores on the test indicates greater frequency of cognitive failures. Due to the absence of a standardized cut-off score for typical aging performance, we used the data from participants in the current study to establish an exclusion threshold. Specifically, we calculated the mean test score for older adults (17.33) and excluded any participants whose score was more than two standard deviations above the mean (i.e., above 34.41).

### Procedure

The experiment consisted of two sessions conducted 7–16 days apart. In the first experimental session, participants completed a brief demographic questionnaire that gathered information about their age, sex, history of language-related disorders, and physical or neurological impairments followed by the Cognitive Failure Questionnaire 2.0 (Goodhew & Edwards, 2024) (older adults only). Participants subsequently completed the picture naming task. The second experimental session consisted of the picture naming task only.

The picture naming task began with a familiarization phase, during which each experimental picture was presented individually on the screen alongside its corresponding target noun. Participants were instructed to study both the image and the associated noun, as they would be required to name these items later in the task. Participants then completed a brief practice phase of 5 trials, followed by the main experimental phase comprising 150 trials. Each trial started with a fixation cross displayed for 500 ms, followed by a picture shown for 2000 ms. After the picture disappeared, a blank screen was presented for 1000 ms. Vocal responses were recorded from the onset of the picture presentation until the end of the trial (i.e, 3000 ms). Participants were asked to name each picture aloud as quickly and accurately as possible. The 150 experimental pictures were presented in a randomized order. All stimuli were presented online using the stimuli presentation software PCIbex (Zehr & Schwarz, 2018).

## Data Preprocessing

Only trials with correct responses were included in the analyses. Incorrect responses included trials in which participants produced an incorrect word, exhibited disfluencies (e.g., false starts), or failed to produce a response within the 3000 ms trial window. The distribution of response types for each group and testing session can be found in Appendix B. The mean naming accuracy of young adults was 95.12% in the first testing session and 95.83% in the second testing session. The mean naming accuracy of older adults was 95.11% in the first testing session and 96.10% in the second testing session. High performance accuracy across groups suggests that our participants performed the experiment in good faith. This is important as previous reports suggest that this is not always the case (e.g., Fairs & Strijkers, 2021). Picture naming latencies were calculated for each trial as the time between the picture onset and the onset of the vocal response; the latter was set manually in Praat (Boersma, 2007). The preprocessed dataset is publicly available at: https://osf.io/7thmy/.

## Analyses

The analyses were conducted using R (R Core Team, 2024) and statistical models were fitted using the R package ‘brms’ (Bürkner, 2017). We conducted analyses in the Bayesian framework whenever possible because, contrary to the frequentist framework, the Bayesian framework enables us to quantify evidence for the null hypothesis (absence of an effect) relative to the alternative hypothesis. For Bayesian analyses we report the mean and 95% credible intervals of the posterior distribution. The 95% credible interval is the interval in which we can be 95% certain that the true value of this effect lies (Gelman & Carlin, 2014). We also report Bayes Factors, which we conducted to quantify the evidence in favor of the alternative over the null hypothesis of no effect. In computing Bayes Factors, we fit null models that are identical to the alternative models, except that they exclude the term of interest (e.g., effect of group). Because Bayes Factors are sensitive to prior specifications, and weakly informative priors tend to favor simpler models, we used more informative priors for the effect of interest when estimating Bayes Factors. To interpret Bayes Factors, we use Lee and Wagenmakers (2014)’s version of Jeffreys (1998)’s scale. We consider values between 1 and 3 as anecdotal evidence, values between 3 and 10 as moderate evidence, values between 10 and 30 as strong evidence, values between 30 and 100 as very strong evidence, and values above 100 to provide extreme evidence.

For the analyses run in the frequentist framework, p-values are used to assess significance, with alpha set to 0.05.

Since data were collected on two separate occasions, we conducted the same analyses for both testing sessions. The analysis script is available in the OSF repository for this study.

### Age group differences in naming latencies

In this analysis we examined whether there is an effect of age on picture naming latencies in our data and we estimate the size of this effect. We fitted a Bayesian hierarchical model predicting naming latency as a function of age group. The random effect structure included by-item intercepts and by-participant intercepts. The by-item intercept accounts for variability in mean naming times across items, while the by-participant intercepts capture inter-individual differences in mean naming latency. In this and following analyses with the predictor group, contrasts were set such that young adults’ data had a value of –0.5 and older adults’ data had a value of 0.5. As such, the intercept represents the grand mean, and a positive estimate indicates slower naming latency for older adults.

We first ran the model with weakly informative priors to obtain the posterior distribution. Weakly informative priors ensure that model outcomes are driven primarily by the data. For the intercept, we used *N*(1000, 200). This prior reflects the assumption that the value for the intercept lies with 95% probability between 600 and 1400 ms, with a higher probability for values around 1000 ms. For the effect of age group, we used *N*(0,200), which reflects the assumption that the effect lies with 95% probability between –400 and 400 ms, with a higher probability for values around 0. We used truncated normal distributions for standard deviations, namely *N*_+_ (0,100) for all random terms and *N*_+_ (0,200) for the residual standard deviation. We then estimated Bayes factors for the effect of age group on naming latencies using the following priors: *N*(0,25), *N*(0,50), *N*(0,100), and *N*(0,150).

### Variability in naming latencies between participants

In this analysis, we examined whether older adults show greater between-participant variability in a picture naming task compared to young adults. We fitted a model that was similar to the one ran in the first analysis (group differences in naming latencies), but included by-participant random intercepts nested within age group. These intercepts reflect each participant’s deviation in mean naming times, relative to the average performance of their group and are adjusted for the effect of item properties. We compared the variance of the by-participant intercepts between groups using a frequentist F-test.

### Variability in naming latencies across experimental items

In this analysis, we tested whether older adults show greater variability in naming latencies across items compared to young adults. To do so, we estimated between-item variability within each group as follows. We fitted a Bayesian hierarchical model predicting naming latency as a function of group. We included a fixed effect for trial number (linear and quadratic) to account for potential practice and fatigue effects. In the random effect structure, we included by-participant intercepts to account for inter-individual differences in mean naming latencies, and by-item intercepts nested under group to obtain an estimation of the variability in mean response times across items for each group. The priors for the intercept and slopes on the mean, as well as for all the standard deviations were identical to those used in the previous analyses. For the effect of trial on the dependent variable, *N*(0,5) reflects the assumption that the effect lies with 95% probability between –10 and 10 ms, with a higher probability for values around 0 ms. We extracted the by-item intercepts for each age group from the model and compared these estimates between groups using a frequentist F-test.

### Variability in naming latencies within participants across trials

In this analysis we tested whether older adults show greater variability within participant across trials compared to young adults. We fitted a Bayesian distributional model to estimate the effect of age group on both mean naming latency and the residual standard deviation (sigma). The model included by-participant and by-item intercepts for both the mean and the residual standard deviation. As such, the residual standard deviation represents the variability left within a participant across trials once the effects of item and participant variability have been accounted for. The priors for the intercept and slopes on the mean were the same as in the previous analyses. The prior for the residual standard deviation was set to *N*_+_ (0,200), and the prior for the effect of age group on the residual standard deviation to *N*_+_ (0,100). According to the latter, the more likely value is 0, with values from 0 to 200 being considered likely. We estimated Bayes factors for the effect of age group on the residual standard deviation using the following priors: *N*(0,25), *N*(0,50), and *N*(0,75).

### Variability in naming latencies within participants across sessions

We examined whether naming latencies varied more within-participant across days for older than younger adults. To do so, we estimated test-retest reliability (the correlation between trials in the first and in the second session), which we then compared between groups.

To estimate test-retest reliability, we fitted a no-intercept Bayesian hierarchical model predicting naming latency as a function of session number (first or second), age, and the interaction between the two variables. The random structure included by-item intercepts, and by-participant intercepts for session number nested within group. As a result of these specifications, the model estimated an intercept for each combination of session number and group. The priors for the slope and the standard deviations were the same as in the previous analyses. To test for group differences in test-retest reliability, we extracted the by-participant intercepts for trials in the first session and in the second session. We then computed correlations between these intercepts and compared them between groups using the R package ‘cocor’ (Diedenhofen & Musch, 2015).^1^

### Power computations

In this last analysis, we ran power simulations to determine the number of participants and items needed to detect an age-related slowing in naming in a simple picture naming task, provided it indeed exists. Simulations in turn require estimates of effect sizes, estimations of between-item and between-participant variability, as well as of residual variability. For our simulations, we used the estimates of the statistical model fitted to test the effect of age group on naming latencies on data in the first testing session. We set the intercept to 1018.08 ms, the age-group effect to 82.83 ms, the between-subject standard deviation to 123.18 ms, the between-item standard deviation to 104.26 ms, and the residual standard deviation to 205.47 ms. We simulated power as a function of the number of participants, from 10 to 100 in steps of 10. We did so for 150 items (number of items in our experiment) and repeated the procedure for 20 to 140 items, in steps of 20. We set statistical power to 80% and the significance level (alpha) to 0.05.

## Results

### Age group differences in naming latencies

**Figure 1:**
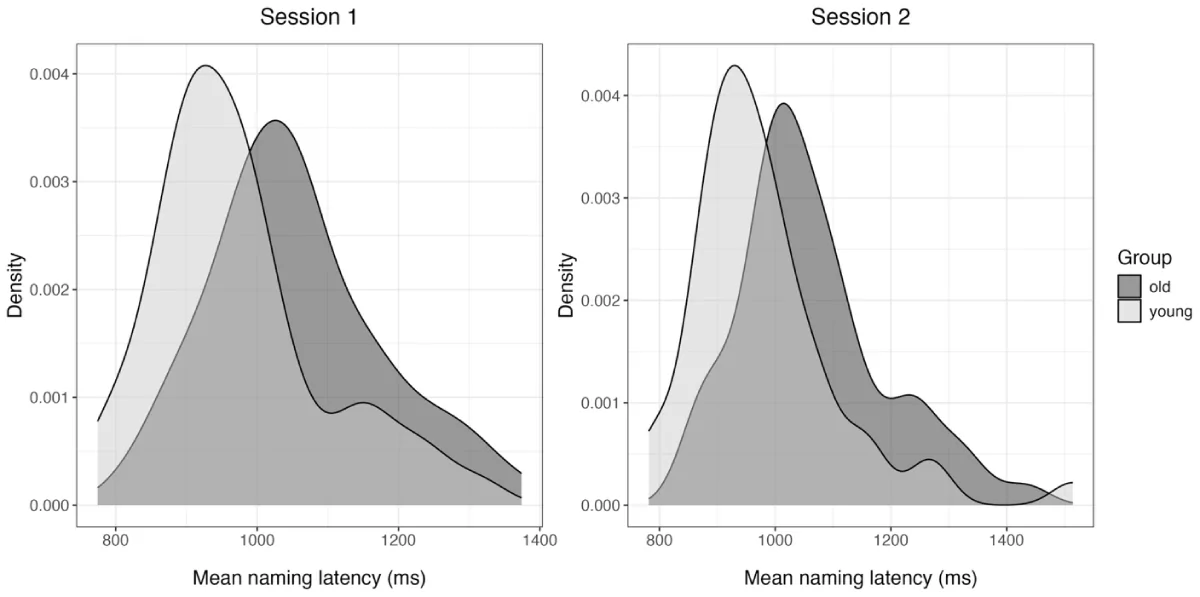
Densities of participants’ mean naming latencies by session and group.

**Figure 2:**
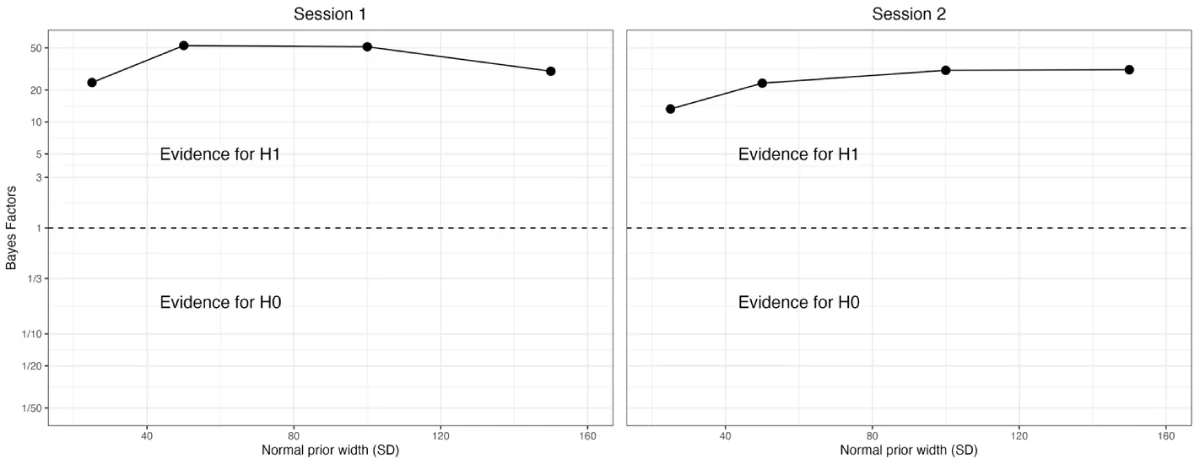
Sensitivity analysis of Bayes factors for age-related slowing in picture naming in the first (left) and second (right) test sessions. *Note*. Data points in the upper half of the figure represent evidence for the hypothesis that older adults exhibit slower naming latencies than younger adults (H1), whereas data points in the lower half represent evidence for the hypothesis that there is no difference in naming latencies between young and older adults (H0).

Figure 1 displays the densities of the participants’ mean naming latencies for young and older adults on the first session (left) and second session (right). Table 2 reports the output of the models testing the effect of group on naming latencies for each session. The results of the Bayes factor analysis are displayed in Figure 2. Older adults exhibited slower naming latencies than younger adults on both testing sessions. For both testing sessions, the Bayes factors provide support for a slowing in naming latencies with age.

**Table 2:** Output of the model testing the effect of age on naming latencies.


SESSION	TERM	ESTIMATE	LOWER	UPPER

Session 1	Intercept	1018.08	991.80	1044.27

	Group	82.83	35.43	131.58

Session 2	Intercept	1023.48	994.99	1052.17

	Group	82.26	31.30	132.37


### Variability in naming latencies between participants

Table 3 reports the estimates of between-participant variability for young and older adults for both testing sessions. For data in the first session, the ratio of the two variances was 1.01 and was not significantly different from 1 (*p* = 0.97). For data in the second session, the ratio of the two variances was 0.93 and was not significantly different from 1 (*p* = 0.80). These results do not provide evidence in support of or against an increase in between-participant variability in naming latencies with age.

**Table 3:** Between-participant variability (variance) for each group and session.


SESSION	GROUP	BETWEEN-PARTICIPANT VARIANCE

Session 1	Old Adults	14800.27

	Young Adults	14607.13

Session 2	Old Adults	15693.62

	Young Adults	16852.63


### Variability in naming latencies across experimental items

Table 4 reports the between-item variability for each group and testing session. Descriptively, older adults exhibited greater between-item variability than younger adults on both testing sessions. For data in the first session, the ratio of the two variances was 1.46 and was significantly greater than 1 (*p* < .001). For data in the second session, the ratio of the two variances was 1.75 and was significantly greater than 1 (*p* < .001). These results indicate that there is greater variability in naming latencies across items in older adults relative to young adults.

**Table 4:** Between-item variability (variance) for each group and session.


SESSION	GROUP	BETWEEN-ITEM VARIABILITY

Session 1	Old Adults	12243.91

	Young Adults	8365.80

Session 2	Old Adults	12656.25

	Young Adults	7229.48


### Variability in naming latencies within participants across trials

**Figure 3:**
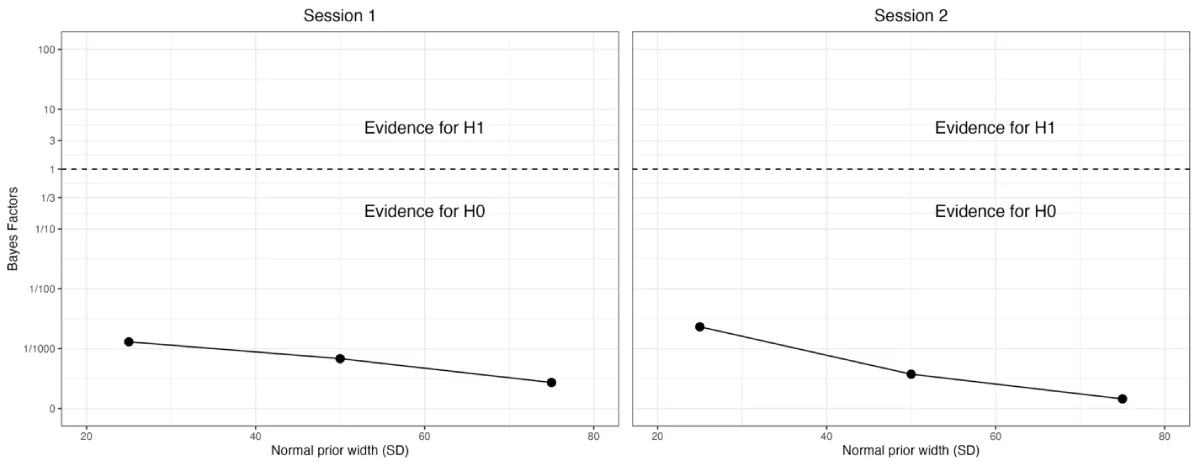
Sensitivity analysis of Bayes factors for the effect of age group on the residual standard deviation in the first (left) and second (right) testing sessions. *Note*. Data points in the upper half of the figure represent evidence for the hypothesis that older adults exhibit greater within-participant variability across trials than young adults (H1), whereas data points in the lower half represent evidence for the hypothesis that there is no difference in within-participant variability across age groups (H0).

Table 5 reports the output of the models testing the effect of group on the residual standard deviation (i.e., variability within individual across trials) for each session. The results of the Bayes factor analyses are displayed in Figure 3. In both sessions, the Bayes factor analysis supports the null hypothesis, indicating no age group differences in the residual standard deviation.

**Table 5:** Output of the model testing the effect of age group on the residual standard deviation (i.e., sigma).


SESSION	TERM	ESTIMATE	LOWER	UPPER

Session 1	Sigma Intercept	5.18	5.11	5.25

	Group	0.01	–0.11	0.13

Session 2	Sigma Intercept	5.19	5.12	5.25

	Group	0.03	–0.09	0.14


### Variability in naming latencies within participants across sessions

In young adults, the correlation between the estimates for the first and second session was 0.82 [0.71, 0.90]. Older adults had a correlation estimate of 0.93 [0.87, 0.96]. Test-retest reliability estimates differed significantly between groups, with older adults showing a significantly higher correlation compared to young adults (*p* = 0.02).

### Power calculations

**Figure 4:**
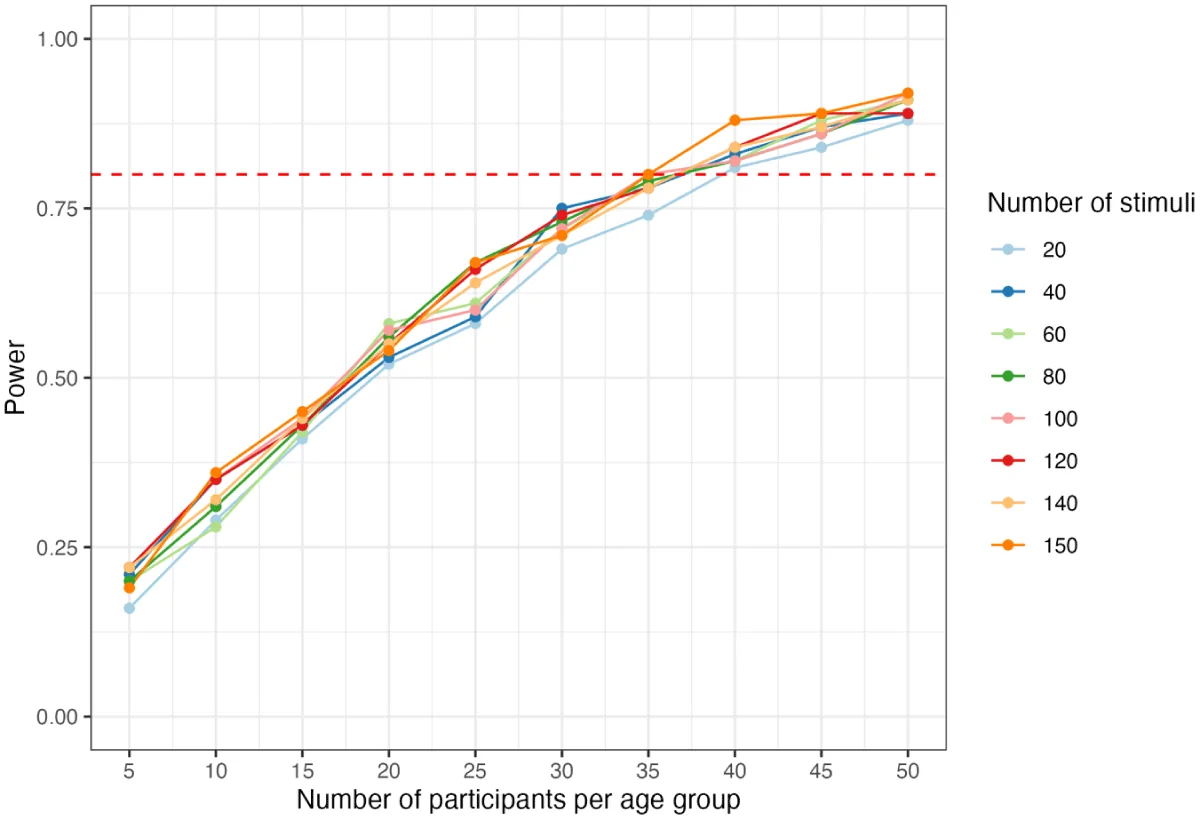
Statistical power to detect an effect of age on naming latencies as a function of the number of participants per age group and the number of items. *Note*. We simulate power for 10 to 100 participants in increments of 10 and for 20 to 140 items in increments of 20, and 150 items. The red line indicates 80% power.

Figure 4 shows the power curve across combinations of item and participant numbers used in our simulations. A table reporting the combinations that achieved at least 80% power is provided in Appendix C. The simulations indicate that we can achieve 80% power with 35 participants per age group and 100 experimental items. Power can also be achieved with fewer items (e.g., 20 items), but this requires a larger participant sample size (40 participants per age group). Adding more items does not seem to increase power substantially: with 35 participants per group, increasing number of items from 100 to 150 keeps power at 80%.

## Discussion

Various forms of performance variability in naming latencies are expected to increase with age, but few studies have examined whether this is the case and, so far, reports of increased variability were often anecdotal. In the present study, we tested whether naming performance becomes more variable with age by comparing young and older age groups in naming latency variability between participants, across items, and within participants across trials and days.

First, we found evidence supporting an age-related difference in naming latencies, with older adults being approximately 80 ms slower in naming pictures compared to young adults (Session 1: 82.83 [35.43, 131.58]; Session 2: 82.26 [31.30, 132.37]). This finding is consistent with previous studies reporting slower naming in old than younger adult groups (Ferré et al., 2020; Hoyau et al., 2017; Krethlow et al., 2024; Oosterhuis et al., 2023; Tsang & Lee, 2003; Verhaegen & Poncelet, 2013; Wierenga et al., 2008). Although the credible intervals around our estimated age-related difference on both testing days indicate some uncertainty regarding the precise magnitude of the effect, they overlap with estimates reported in several previous studies (e.g., 109 ms in Krethlow et al., 2024; 133 ms in Ferré et al., 2020; 117 ms in Oosterhuis, et al., 2023; 99 ms in Hoyau et al., 2017; but see Tsang & Lee, 2003; Verhaegen & Poncelet, 2013; and Wierenga et al., 2008 for larger effect sizes).

Second, we found no evidence for or against an age-related increase in between-participant variability. Between-participant variability in mean naming latencies (operationalised as the variance of the by-participant intercepts) was descriptively similar for the two age groups (Session 1: older adults = 14800 ms², young adults = 14607 ms²; Session 2: older adults = 15694 ms², young adults = 16853 ms²). This finding contrasts previous studies reporting greater variability in naming performance of older than younger adults (Ferré et al., 2020; Oosterhuis et al., 2023; Tsang & Lee, 2003) However, these previous studies did not test statically for a difference in variability, making it impossible to determine whether the reported greater variability reflects reliable characteristics of age-related changes in naming performance. Our finding suggests that, at least in our sample, the age-related slowing was not accompanied by greater heterogeneity between individuals.

We note however, that a few of our young participants had very slow naming latencies compared to the mean naming latencies of other participants in the same group (refer to the density plot of participants’ mean naming latencies, Figure 1), which likely influenced the outcome of our analysis. Unfortunately, we do not have any mean of determining whether these young participants represent outliers or show typical naming times for that group. Future studies using larger samples of young adults are needed to better characterize the distribution of naming latencies in this age group.

Third, we found increased between-item variability in older adults. This finding is in line with previous reports showing that stimulus properties have a greater influence on naming latencies in older than younger adults (LaGrone & Spieler, 2006; Spieler & Balota, 2000). In particular, previous studies have suggested that older speakers are more sensitive to word frequency and name agreement. However, these studies did not control for other possible lexical properties, or for differences in the visual properties of the picture stimuli. Identifying which lexical properties increase variability in older adults is an important direction for future research, as this may provide insight into the mechanisms underlying age-related changes in word retrieval. For instance, greater effects of name agreement in older adults were interpreted as reflecting an age-related decline in the efficiency of lateral inhibitory connections between competing lemmas (LaGrone and Spieler, 2006). More generally, the greater between-item variability in older adults raises the possibility that differences across studies in the magnitude of the age-related slowing in latencies may partly reflect differences in the items included in the stimulus sets.

With respect to variability across trials, we found evidence for no increases in within-participant variability with age. Greater variability in older adults’ performance has been argued to arise in tasks that more heavily rely on executive functions relatively to tasks that tax on these processes to a smaller extent (West et al., 2002). The picture naming task we used was relatively simple, and we speculate that executive functions are not sufficiently recruited in such a task. Increases in within-participant variability with age are more likely to be observable in more complex naming tasks that more heavily rely on executive functions. Future studies could test whether within-participant variability increases with age in tasks that more heavily rely on executive functions.

With respect to variability across days, while latencies of both age groups showed excellent test-retest reliability, reliability of older adults’ latencies was higher than that of young adults. A previous study testing test-retest reliability of older adults’ latencies found a smaller correlation estimate (i.e., *r* = 0.77, Hamberger, Heydari, Caccappolo, & Seidel, 2022) than the one we found, but this may have been due to the correlation being estimated without accounting for item properties, as this approach may underestimate the correlation. The rather high test-retest reliability in both groups suggests that one session is probably enough to test for age group differences. We further note that our estimates of test-retest reliability and their credible intervals are useful to estimate the sample sizes for studies that plan on correlating the naming latencies of older adults with other measures (such as executive control, see for instance Shao, Roelofs, & Meyer, 2012).

Our power calculations showed that 80% power can be achieved with 35 participants per age group and 100 items. The simulations suggest that increasing the number of participants has a greater impact on power than increasing the number of items. This is to be expected in a between group comparison (Westfall, Kenny, & Judd, 2014). Moreover, and as demonstrated by Westfall et al. (2014), statistical power can be improved by increasing the sample size of the random factor that contributes the greatest amount of random variation to the data. In the present study, the estimated standard deviations of the by-participant random intercepts were larger than those of the by-item intercepts, indicating that participants contributed more variability to naming latencies than items.

We have argued that examining age-related differences in the effect of item properties on naming latencies may provide insights into the mechanisms underlying age-related changes in word retrieval. Addressing this issue will require designing studies which directly compare the effects of stimulus properties or experimental manipulations across age groups. Our power simulations do not inform on the sample sizes needed for such studies. Importantly, however, our finding that older participants show greater between-item variability compared to young adults highlights the importance of considering item sample sizes in studies of aging. Specifically, sample sizes that are adequate for investigating effects in young adults may not be sufficient to account for the greater variability present in older adults.

We note that the results of our power analysis depend on the estimates we specified for the size of the effect of age on naming latencies and for the various types of variability. In our analysis, the credible interval of our model (35.43–131.58) shows that the effect of age on latencies may be much smaller than the estimate we chose for the simulation (i.e., 82.83 ms). This means that we may need larger samples of participants to achieve sufficient power. Finally, we note that the results of our power analysis are only valid for the age groups considered in this study (i.e., young adults: 18–35 and older adults: 65–75).

## Additional File

The additional file for this article can be found as follows:

10.5334/joc.520.s1 Appendices. Appendix A to C.

## Data Availability

Our ethics board does not allow us to make raw audio recordings of participants publicly available. However, the preprocessed data set includes the raw output from the experimental software (participant number, trial number, item), as well as the response time and accuracy for each trial. We additionally share participants’ demographic information (age, sex) and scores on the Cognitive Failure Questionnaire 2.0 (Goodhew & Edwards, 2024) for older adults. These data are available in the OSF repository for this project: (DOI: 10.17605/OSF.IO/7THMY).

## Note

It is possible to estimate the correlation of random effects in a Bayesian hierarchical model directly, as done in Fuhrmeister et al. (2024) and detailed in Chen et al. (2021), but this approach does not allow comparing correlations across groups. To compare correlation estimates between groups, we extracted participant intercepts for each trial type. We computed frequentist correlations with these intercepts as we are not aware of a way of comparing correlations in the Bayesian framework.
