# Supplementary material for: Age-Related Differences in Naming Latency Variability
Source: J Cogn. 2026 Sep 21;9(1):48. doi: 10.5334/joc.520 (PMC13614249; doi:10.5334/joc.520)
Supplement: Supplementary file 1 [file joc-9-1-520-s1.pdf]

Appendix A

List A						
anchor	camel	dummy	judge	onion	saw	tiger
apple	candle	ear	kangaroo	orange	scissors	tomato
arm	carrot	elbow	key	owl	shield	tongue
arrow	castle	fairy	kitchen	pear	shoe	tractor
avocado	caterpillar	fence	kite	peg	shower	train
basket	cherry	fireplace	kiwi	pencil	skirt	tray
battery	circle	flag	knife	pepper	snake	tree
beak	clown	flipper	koala	piano	soap	triangle
bear	coat	flower	lamp	pineapple	squirrel	tunnel
bell	comb	fork	lawnmower	pipe	star	wall
bench	compass	fox	lighter	plumber	submarine	wave
bib	computer	fridge	lighthouse	pocket	suit	whale
bike	cork	ghost	limo	potato	suitcase	wheelbarrow
bone	cow	glove	lung	puppet	sun	windmill
book	crab	goat	magnet	queen	swan	wing
bottle	curtain	gym	map	rabbit	sweet	zebra
boxer	dice	hat	medal	rhino	sword	zip
bracelet	doctor	heel	mermaid	robot	tambourine	
bull	donkey	helicopter	mountain	rocket	tap	
bullet	doughnut	helmet	mouth	rope	tattoo	
bus	drawer	hippo	nest	rose	teacher	
butter	dress	island	net	ruler	thumb	
butterfly	drum	jellyfish	olive	sausage	tie	

List B						
airport	burger	crown	foot	knee	pool	strawberry
ambulance	butcher	dentist	football	lab	printer	swimming
ankle	button	desert	frog	leaf	pumpkin	tank
apron	cactus	devil	fruit	leg	ring	telephone
asparagus	cage	diamond	giraffe	lemon	road	television
baby	calculator	dinosaur	girl	lion	roof	thermometer
back	calendar	dog	glass	lizard	scarecrow	tooth
balloon	camera	dolphin	glasses	man	scarf	torch
banana	cannon	dominoes	greenhouse	mask	scorpion	trumpet
bat	car	door	guitar	maze	screwdriver	umbrella
beard	caravan	dragon	gun	microphone	shadow	vein
bed	chain	duck	hair	mirror	shark	volcano
bedroom	chair	earring	hand	moustache	sheep	waiter
belt	chocolate	egg	handle	needle	shirt	wallet
bible	choir	elephant	harp	nose	sink	watch
bin	cigarette	eye	heart	nun	skateboard	well
bomb	cloud	face	hedgehog	nurse	skeleton	witch
boomerang	coconut	fan	honey	parachute	skull	
box	coffee	farm	house	parrot	snail	
bra	coffin	feather	iron	penguin	sock	
brain	condom	finger	jar	pilot	spider	
broccoli	cone	fire	keyboard	pirate	spoon	
broom	cowboy	fist	king	pizza	stapler	

Appendix B

Distribution of participants' response types by group and session (in percentages).

Session	Group	Trial Type	Count	Percentage (%)
1	old	correct	7658	95.11
		disfluency	60	0.75
		no response	106	1.32
		incorrect word	228	2.83
	young	correct	7134	95.12
		disfluency	41	0.55
		no response	93	1.24
		incorrect word	232	3.09
2	old	correct	7784	96.10
		disfluency	37	0.46
		no response	78	0.96
		incorrect word	201	2.48
	young	correct	7187	95.83
		disfluency	48	0.64
		no response	50	0.67
		incorrect word	215	2.87

Appendix C

Number of participants per age group and number of items needed to reach at least 80% power in a picture naming study comparing young (18–35) and older adult (65–75) groups

Number of participants per age group	Number of items	Power
35	100	0.80
35	150	0.80
40	20	0.81
40	40	0.83
40	60	0.82
40	80	0.82
40	100	0.82
40	120	0.84
40	140	0.84
40	150	0.88
45	20	0.84
45	40	0.87
45	60	0.88
45	80	0.86
45	100	0.86
45	120	0.89
45	140	0.87
45	150	0.89
50	20	0.88
50	40	0.89
50	60	0.91
50	80	0.91
50	100	0.92
50	120	0.89
50	140	0.91
50	150	0.92
